# Magnetic resonance imaging findings after stereotactic body radiation therapy for hepatocellular carcinoma - a scoping literature review

**DOI:** 10.1016/j.ctro.2026.101227

**Published:** 2026-07-06

**Authors:** Caglayan Lara, Gkika Eleni, Péporté Anne, Stocker Daniel, Ghafoor Soleen

**Affiliations:** a Department of Radiation Oncology, University Hospital Bonn, Bonn, Germany; b Department of Radiology, Cantonal Hospital Frauenfeld, Frauenfeld, Switzerland; c Diagnostic and Interventional Radiology, University Hospital Zurich, University of Zurich, Zurich, Switzerland

**Keywords:** HCC, MRI, SBRT

## Abstract

Stereotactic body radiotherapy (SBRT) is an established locoregional treatment option for hepatocellular carcinoma (HCC). However, post-treatment imaging remains challenging because tumor response and focal liver reaction (FLR), defined as radiation-related alterations of the adjacent liver parenchyma, may both evolve gradually and mimic viable or recurrent tumor.

We conducted a scoping review in accordance with the Preferred Reporting Items for Systematic Reviews and Meta-Analyses extension for Scoping Reviews (PRISMA-ScR). PubMed, Embase, and manual reference screening were used to identify studies published between January 2012 and February 2025 that evaluated MRI findings after SBRT for HCC. Studies were eligible if they reported MRI-based post-treatment outcomes after stereotactic, image-guided, highly conformal external-beam radiotherapy delivered in a limited number of fractions with ablative intent. Studies evaluating non-SBRT regimens, non-MRI imaging modalities, theoretical modeling approaches, reviews, and case reports were excluded.

Eight studies met the inclusion criteria. Overall, MRI findings after SBRT demonstrated a delayed and time-dependent pattern rather than immediate tumor regression. In responding lesions, arterial phase hyperenhancement (APHE) and washout commonly decreased over time or evolved toward delayed enhancement or non-enhancement. T2-weighted and diffusion-weighted imaging (DWI) signal intensity tended to decline, whereas apparent diffusion coefficient (ADC) values increased. FLR was frequently observed and included perilesional enhancement, hepatobiliary phase hypointensity, capsular retraction, and dose-related parenchymal changes on gadoxetic acid–enhanced MRI.

Response assessment after SBRT should therefore rely on serial multiparametric MRI rather than early arterial enhancement alone. Persistent enhancement shortly after treatment should not automatically be interpreted as treatment failure, and FLR remains an important source of diagnostic uncertainty. Prospective studies are needed to standardize MRI protocols, follow-up intervals, and response assessment criteria in this setting.

## 1 Introduction

Hepatocellular carcinoma (HCC) is the most common primary liver malignancy, and many patients present with tumor stage, comorbidities, or impaired liver function that preclude curative-intent surgery [1] As a result, locoregional therapies are central to disease management. Stereotactic body radiation therapy (SBRT) has emerged as an important option, particularly for patients who are not candidates for resection or thermal ablation and in whom transarterial therapies are unsuitable, have failed, or are contraindicated [2]. The goal of liver SBRT is to deliver a highly conformal, ablative dose to the tumor while sparing adjacent non-tumorous liver parenchyma and nearby organs at risk [3]. Reported 1-year local control rates are high (approximately 87–92%), with outcomes influenced by radiation dose, tumor characteristics, and baseline liver function [4], [5]. Contemporary clinical guidelines e.g. NCCN (National Comprehensive Cancer Network), ESMO (European Society for Medical Oncology ands ASTRO (American Society for Radiation Oncology) increasingly recognize SBRT as a locoregional treatment option in selected patients with preserved liver function, including those with early-stage disease who are not suitable for ablation or who develop intrahepatic recurrence after prior locoregional therapy [6], [7]. Despite favorable local control, imaging-based response assessment after SBRT remains challenging. Post-radiation changes in lesion size and enhancement evolve gradually and may take months to stabilize, limiting the usefulness of size-based criteria alone **[3]**. Moreover, radiation-induced changes in the surrounding liver parenchyma can produce focal enhancement and signal alterations that mimic residual or recurrent tumor, creating important interpretive pitfalls. In routine practice, response assessment after radiation-based therapy relies on multiparametric imaging-most commonly contrast-enhanced MRI and CT-integrating changes in enhancement patterns, non-enhancing components, and diffusion metrics. Standardized frameworks include mRECIST, which defines viable tumor primarily by arterial phase hyperenhancement (APHE), and the LI-RADS treatment response algorithm, which additionally incorporates washout and characteristic post-treatment enhancement patterns. For post-treatment response assessment, mRECIST (modified Response Evaluation Criteria in Solid Tumors) and the LI-RADS (Liver Imaging Reporting and Data System) treatment response algorithm represent two commonly used standardized frameworks. mRECIST evaluates residual viable tumor mainly on the basis of arterial phase hyperenhancement (APHE), whereas LI-RADS applies a broader imaging approach by also considering washout and characteristic post-treatment enhancement patterns. The 2024 LI-RADS update further refined this algorithm by providing dedicated guidance for response assessment after radiation-based locoregional therapies [8], [9], [10], [11].

For consistency, this review uses the term focal liver reaction (FLR) to describe radiation-related parenchymal changes within or adjacent to the treated liver region, including perilesional enhancement, T2-weighted hyperintensity, hepatobiliary phase hypointensity, capsular retraction, and focal volume loss.

In clinical practice, response assessment after SBRT is typically multiparametric, integrating changes in lesion size and enhancement patterns as well as the extent of non-enhancing (presumed necrotic) tumor components on MRI and CT [12]. However, interpretation remains challenging after radiation-based locoregional therapy and is prone to misclassification. Given the evolving post-SBRT MRI appearances and the risk of misclassification, this PRISMA-ScR–guided scoping review aims to map and synthesize the available evidence on MRI findings in treated HCC lesions and the adjacent liver parenchyma after SBRT, with particular emphasis on temporal evolution and imaging features that may support more standardized and accurate response assessment.

## 2 Methods

**Fig. 1:**
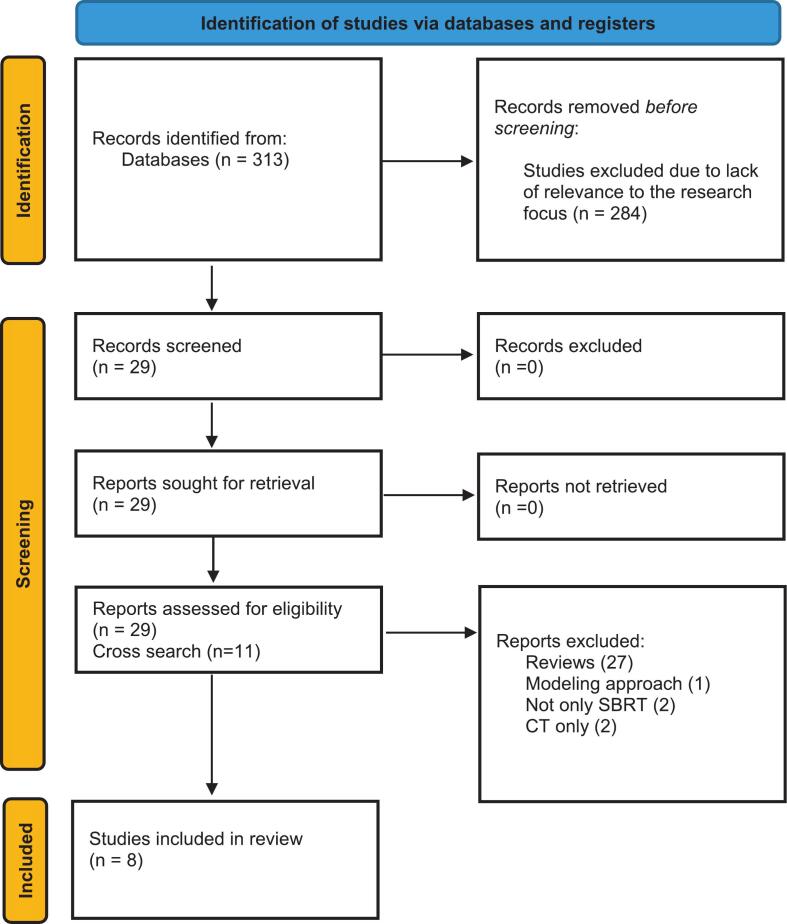
PRISMA [23] flow diagram of study selection. Abbreviations: SBRT: Stereotactic body radiation therapy; CT = computed tomography.

This scoping review was conducted in accordance with the Preferred Reporting Items for Systematic Reviews and Meta-Analyses extension for Scoping Reviews (PRISMA-ScR) (Fig. 1; Table 1). We searched PubMed (MEDLINE) and Embase, supplemented by manual cross-referencing, to identify studies published from January 2012 to February 2025 evaluating MRI findings after SBRT for HCC. Searching and study selection were performed independently by a radiation oncology resident (L.C., 4 years of experience) and a senior abdominal radiologist (S.G., 12 years of experience). In PubMed, the following strategy was used: ((“Hepatocellular Carcinoma”[Mesh] OR hepatocellular carcinoma[tiab] OR HCC[tiab] OR liver cancer[tiab] OR “Liver Neoplasms”[Mesh]) AND (“Stereotactic Body Radiotherapy”[Mesh] OR stereotactic body radiotherapy[tiab] OR SBRT[tiab] OR stereotactic ablative radiotherapy[tiab] OR SABR[tiab] OR stereotactic radiation[tiab]) AND (“Magnetic Resonance Imaging”[Mesh] OR magnetic resonance imaging[tiab] OR MRI[tiab] OR MR[tiab] OR MR imaging[tiab] OR magnetic resonance[tiab] OR gadoxetic[tiab] OR gadoxetate[tiab] OR EOB[tiab] OR Primovist[tiab] OR imaging[tiab] OR radiology[tiab])). Eligible studies included adults with HCC treated with SBRT, which was operationally defined for this review as stereotactic, image-guided, highly conformal external-beam radiotherapy delivered in a limited number of fractions with ablative intent. A fraction dose >4 Gy was used only as a minimum screening criterion when accompanied by a clearly described stereotactic technique; conventional or non-stereotactic hypofractionated regimens were excluded. Eligible studies were required to report post-treatment multiparametric MRI findings of treated lesions and/or adjacent liver parenchyma, including dynamic contrast-enhanced imaging, diffusion-weighted imaging (DWI), hepatobiliary phase imaging, or magnetic resonance elastography.

**Table 1:** Characteristics of Included Studies (PRISMA-Aligned). Abbreviations: **ADC** = apparent diffusion coefficient; **AFP** = alpha-fetoprotein; **CT** = computed tomography; **DWI** = diffusion-weighted imaging; **Gd-EOB-DTPA** = gadolinium–ethoxybenzyl–diethylenetriamine pentaacetic acid; **HCC** = hepatocellular carcinoma; **LI-RADS** = Liver Imaging Reporting and Data System; **mRECIST** = modified Response Evaluation Criteria in Solid Tumors; **MRE** = magnetic resonance elastography; **MRI** = magnetic resonance imaging; **RECIST** = Response Evaluation Criteria in Solid Tumors; **RT** = radiation therapy; **SBRT** = stereotactic body radiation therapy; **T1WI** = T1-weighted imaging; **T2WI** = T2-weighted imaging; **TRA** = treatment response assessment; **D50** = dose delivered to 50% of the target volume.

Study	Study Design	Study Period	Participants (n)	Target Condition	Index Test(s)	Reference Standard	Outcomes Assessed
Mai et al., 2023 (15)	Retrospective cohort	Jan 2015–Dec 2020	102 patients (102 HCCs)	SBRT-treated HCC	Multiparametric MRI (T1WI, T2WI, DWI, dynamic contrast, hepatobiliary phase)	Imaging follow-up with lesion growth criteria; AFP trends	Temporal evolution of MRI features; local progression vs non-progression; LI-RADS TRA performance
Mendiratta-Lala et al., 2018 (14)	Retrospective case series	2007–2015	10 patients (10 HCCs)	SBRT-treated HCC	MRI or multiphasic CT	Explant pathology (>90% necrosis) or AFP normalization	Post-SBRT enhancement patterns in confirmed responders
Oldrini et al., 2017 (15)	Retrospective cohort	Oct 2011–Apr 2013	27 patients (35–48 HCCs)	SBRT-treated HCC	MRI (dynamic contrast, T2WI, DWI)	Imaging-based RECIST and mRECIST	Local response rates; predictors of response; MRI response patterns
Yu et al., 2014 (19)	Retrospective cohort	Jul 2011–Feb 2013	48 patients	RT-treated HCC (including SBRT)	DWI with ADC quantification	Local progression-free survival; mRECIST	Association between ADC change and treatment response
Serafini et al., 2022 (17)	Retrospective cohort	Jan 2013–Nov 2019	49 patients (61 HCCs)	SBRT-treated HCC	Gadoxetic acid–enhanced MRI (including hepatobiliary phase)	Imaging follow-up (infield vs outfield progression)	MRI response features; focal liver reaction; progression patterns
Omiya et al., 2023 (18)	Retrospective cohort with control group	Jan 2015–Dec 2020	22 patients; 27 controls	SBRT-treated HCC	MRI with MRE and DWI	Quantitative MRI changes by radiation dose region	Dose-dependent liver stiffness and ADC changes
Nakamura et al., 2015 (20)	Retrospective cohort	Dec 2010–2013	30 patients	SBRT-treated HCC	Gadoxetate-enhanced MRI (hepatobiliary phase metrics)	Change in Child–Pugh score at 6 months	Prediction of post-SBRT hepatic function decline
Jung et al., 2016 (21)	Retrospective dose–response analysis	Mar 2007–Dec 2012	17 patients	SBRT-treated HCC	Gd-EOB-DTPA–enhanced MRI	Dose–signal correlation (D50)	Threshold radiation dose for hepatobiliary phase parenchymal change

Studies were required to report a reference standard for response assessment or functional change (e.g., imaging follow-up, clinical outcomes, histopathology, or biochemical markers). We excluded studies using hypofractionated or conventional fractionation regimens not consistent with SBRT, studies focused on non-MRI modalities, theoretical/modeling work without clinical imaging data, and publications that were reviews, case reports, non-original research, non-English articles, or otherwise not reporting original clinical outcomes. Titles and abstracts were screened followed by full-text review; discrepancies were resolved by consensus. No review protocol was registered. Data extraction was performed independently using a standardized form capturing study design, population characteristics, SBRT regimens, MRI techniques and timing, reference standards, and reported imaging findings. Given heterogeneity in study designs, MRI protocols, follow-up intervals, and outcome definitions, no quantitative synthesis was undertaken; results were summarized narratively by imaging feature category. Consistent with PRISMA-ScR guidance, studies were not excluded based on methodological quality; however, methodological considerations were assessed descriptively using a modified QUADAS-2 framework to contextualize the evidence **(**Table 2**)**.

**Table 2:** Risk of Bias and Applicability Assessment (QUADAS-2–Adapted).

Study	Patient Selection	Index Test (MRI)	Reference Standard	Flow & Timing	Applicability Concerns
Mai et al., 2023 (16)	Low	Low	Moderate	Low	Low
Mendiratta-Lala et al., 2018 (14)	High	Low	Low	Moderate	Low
Oldrini et al., 2017 (15)	Moderate	Low	Moderate	Low	Low
Yu et al., 2014 (19)	Moderate	Low	Moderate	Low	Moderate
Serafini et al., 2022 (17)	Moderate	Low	Moderate	Low	Low
Omiya et al., 2023 (18)	Moderate	Low	Moderate	Low	Low
Nakamura et al., 2015 (20)	Moderate	Low	Low	Low	Low
Jung et al., 2016 (21)	High	Low	Moderate	High	Moderate

After duplicate removal, title/abstract screening and full-text assessment were performed independently by the two reviewers (L.C. and S.G.); records considered potentially eligible by either reviewer proceeded to full-text review, and disagreements were resolved by consensus. Data extraction used a standardized form covering study design, patient population, SBRT regimen, MRI protocol and timing, reference standard, and reported imaging findings. The modified QUADAS-2 [13] framework assessed patient selection, MRI index test, reference standard, flow/timing, and applicability, with each domain rated descriptively as low, moderate, or high risk/concern and used for contextualization rather than study exclusion.

## 3 Results

### 3.1 Study characteristics and methodological considerations

Most included studies were retrospective, with potential patient selection bias related to the availability of follow-up MRI. MRI follow-up was performed at regular intervals, most commonly every 3 months during the first year after SBRT (e.g., at 3, 6, 9, and 12 months), with subsequent imaging every 3–6 months. For example, Mendiratta-Lala et al. [14] reported follow-up imaging at 3, 6, 9, and 12 months after SBRT. Oldrini et al. [15] planned MRI examinations every 3 months during the first two years after treatment. Similarly, Mai et al. [16] scheduled MRI examinations at 1, 3, 6, 9, and 12 months after SBRT, followed by imaging every 3–6 months during the subsequent follow-up period. Risk of index test bias was generally low because MRI acquisition protocols and interpretation criteria were prespecified and applied consistently within studies. Reference standards relied predominantly on imaging follow-up and clinical parameters rather than histopathology, resulting in moderate risk of reference standard bias. Flow and timing bias was limited overall, although some studies assessed predefined post-SBRT time points. Applicability concerns were low, as patient populations, SBRT approaches, and MRI techniques reflected routine clinical practice (Table 3).

**Table 3:** Summary of post-SBRT imaging features of key studies. Abbreviations: **APHE** = arterial phase hyperenhancement; **DWI** = diffusion-weighted imaging; **ADC** = apparent diffusion coefficient; **FLR** = focal liver reaction; **AFP** = alpha-fetoprotein; **MRE** = magnetic resonance elastography; **LSC** = liver–spleen contrast; **Gy** = gray.

Imaging Feature	Typical Post-SBRT Findings	Temporal Evolution	Association With Response / Outcome	Key Studies
Contrast enhancement (APHE, washout)	Progressive loss of APHE and washout; transition to delayed enhancement or non-enhancement; persistent APHE/washout may occur despite response	APHE decreases over 3–12 months; delayed enhancement or non-enhancement stabilizes by ∼6–9 months	Persistent APHE/washout associated with progression in some cohorts, but may persist without growth or AFP elevation	Mai et al.16; Mendiratta-Lala et al.14; Oldrini et al.15
Perilesional enhancement	Ring-like or peripheral arterial enhancement reflecting focal liver reaction (FLR)	Common early (≈3 months) and may persist up to 12 months	Not associated with local progression; considered benign post-radiation change	Oldrini et al.15; Serafini et al.17; Omiya et al.18
T1-weighted signal	Increased intralesional T1 signal intensity	Increases within first months; stabilizes by 6–9 months	Associated with treatment response	Mai et al.16
T2-weighted signal	Decreasing intralesional T2 hyperintensity; frequent perilesional T2 hyperintensity	Intralesional decrease over time; perilesional hyperintensity may persist	Absence of T2 hyperintensity associated with higher response rates; increased T2 suggests progression	Mai et al.16; Oldrini et al.15; Serafini et al.17; Omiya et al.18
DWI signal	Reduction in DWI hyperintensity	Progressive decline during follow-up	Absence of DWI hyperintensity associated with response; increased DWI signal suggests progression	Mai et al.16; Oldrini et al.15; Serafini et al.17
ADC values	Significant post-treatment increase in ADC	Detectable early after treatment	≥20% ADC increase associated with improved local progression-free survival	Yu et al.19; Serafini et al.187 Omiya et al.18
Hepatobiliary phase	Band-like hypointensity in irradiated liver parenchyma (FLR)	Appears within ∼4–6 months; persists long-term	Reflects radiation-induced hepatocyte dysfunction; dose-dependent	Serafini et al.17; Omiya et al.18;
Functional hepatobiliary metrics	Reduced liver–spleen contrast (LSC) in high-dose regions	Correlates with radiation dose (≥30 Gy)	Pre-treatment LSC predicts post-SBRT hepatic function decline	Nakamura et al.20
Other features (MRE, morphology)	Increased liver stiffness on MRE in high-dose regions; capsular retraction	Stiffness increases by ∼4 months; capsular retraction progresses up to 12 months	Reflects radiation-induced fibrosis and parenchymal remodeling	Oldrini et al.15; Serafini et al.17; Omiya et al.18

### 3.2 Contrast enhancement patterns (APHE and washout)

**Fig. 2:**
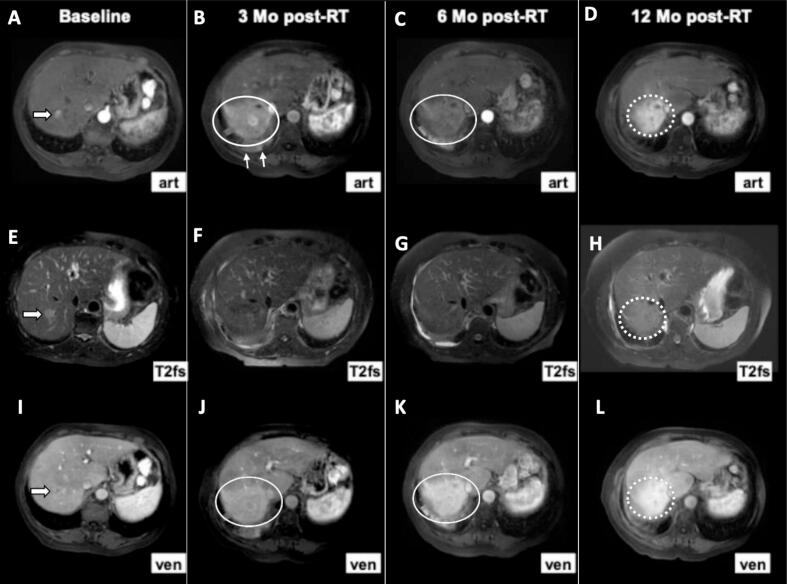
Multiparametric MRI appearance of HCC and surrounding liver parenchyma after SBRT. Axial T1-weighted contrast-enhanced arterial phase (A–D), axial T2-weighted fat-suppressed (E–H), and axial T1-weighted contrast-enhanced portal venous phase (I–L) images. The baseline examination shows a T2-weighted hyperintense lesion in segment VII with non-rim arterial hyperenhancement (APHE) and portal venous washout (arrows in A, E, I), typical for HCC. After SBRT, serial MRI demonstrates temporal and spatial changes that help differentiate treatment response from local tumor progression and benign radiation-induced effects. Within the treated lesion, early post-treatment findings include persistent APHE (B) and washout (J), followed by gradual conversion to delayed enhancement or non-enhancement (D, K, L), with increasing T1-weighted and decreasing T2-weighted signal (F—H). In contrast, local progression would show persistent or increasing, nodular APHE with washout and increased T2-weighted signal. A focal liver reaction surrounding the lesion is frequently seen, typically manifesting as ring-like or perilesional arterial enhancement (B—D) and diffuse T2-weighted hyperintensity (F—H) eventually leading to volume loss and retraction of the surrounding parenchyma; these perilesional and parenchymal changes may persist for months and should not be mistaken for residual or recurrent tumor. Note also the adjacent radiation-induced lung atelectasis next to segment VII (arrow in B). Abbreviations: art = arterial phase, ven = portal venous phase, T2fs = T2-weighted fat-suppressed.

Across studies, post-SBRT enhancement demonstrated a characteristic time-dependent evolution (Fig. 2). In non-progressing lesions, Mai et al. [16] reported that baseline APHE and washout gradually transitioned to delayed enhancement in 71.9% of cases and to complete non-enhancement in 20.8%, typically stabilizing after 6–9 months; by contrast, all progressive lesions showed persistent APHE and washout.

Similarly, Oldrini et al. [15] observed a progressive decline in APHE, with loss of APHE in 54.3% of lesions at 3 months and 74.3% at 6 months. However, persistent enhancement early after SBRT was also frequently reported despite confirmed response: Mendiratta-Lala et al. [14] observed central APHE in 40% and washout in 90% of lesions within the first year, despite evidence of response (≥90% necrosis on explant pathology or AFP normalization), absence of interval growth, and size reduction in 90% of lesions.

Perilesional enhancement was common and often interpreted as FLR rather than residual tumor. Oldrini et al. [15] reported ring-like enhancement in 97.1% of lesions at 3 months, persisting in 82.2% at 12 months. Serafini et al. [17] similarly described ring-like enhancement in 82% and 69% of lesions at approximately 4 and 9 months, respectively. Omiya et al. [18] reported perilesional APHE in 63% of patients at a median of 8 months, attributed to FLR.

### 3.3 T1- and T2-weighted signal intensity

Conventional sequence signal changes were consistent across studies and generally aligned with treatment response. Mai et al. [16] reported increasing T1-weighted signal intensity in 99% of non-progressing lesions and decreasing T2-weighted signal intensity in 92.9%, with stabilization after 6–9 months; progressive lesions showed increasing T2-weighted signal intensity. Oldrini et al. [15] likewise observed a decline in T2 hyperintensity over time, and absence of T2 hyperintensity was associated with higher response rates (*p* = 0.006). Serafini et al. [17] reported a reduction in T2 hyperintensity from 62% at baseline to 30% at first follow-up (∼4 months; *p* = 0.0006).

Peritumoral T2 hyperintensity was frequently described as.a component of FLR. Serafini et al. [17] observed perilesional T2 hyperintensity in 84% at ∼4 months and 75% at ∼9 months, while Omiya et al. [18] reported T2 hyperintensity in 81% at a median of 13 months, consistent with FLR.

### 3.4 Diffusion-weighted imaging and apparent diffusion coefficient

DWI findings consistently reflected response-related tissue changes. Mai et al. [16] reported decreasing DWI signal intensity in 99% of non-progressing lesions, whereas progressive tumors showed increased DWI signal. Oldrini et al. [15] found that absence of DWI hyperintensity was associated with higher response rates (*p* = 0.039).

Quantitative diffusion metrics supported these observations. Yu et al. [19] demonstrated a significant increase in mean ADC values following radiotherapy (from 1.21 × 10^−3^ to 1.41 × 10^−3^ mm^2^/s; *p* < 0.001), with an ADC increase ≥20% predicting superior local progression-free survival (PFS). Serafini et al. [17] reported decreasing DWI hyperintensity between ∼4 and ∼ 9 months (68% to 18%, *p* < 0.0001) with a concomitant rise in median ADC values (1.01 to 1.38 × 10^−3^ mm^2^/s; p < 0.0001). Omiya et al. [18] observed ADC increases in liver regions receiving >30 Gy (1.10 to 1.40 × 10^−3^ mm^2^/s; *p* < 0.05), interpreted as dose-related parenchymal effects rather than tumor-specific response.

### 3.5 Hepatobiliary phase imaging

Hepatobiliary phase abnormalities were consistently reported as part of FLR-related functional alteration in irradiated liver parenchyma. Serafini et al. [17] described band-like hepatobiliary phase hypointensity in 95% of patients at ∼4 months and 94% at ∼9 months, attributed to FLR. Omiya et al. [18] observed hepatobiliary phase hypointensity in irradiated regions in all patients at a median of 4 months.

Several studies emphasized dose dependence. Nakamura et al. [20] demonstrated reduced liver–spleen contrast (LSC) in regions receiving ≥30 Gy, consistent with impaired hepatocyte function; volume-weighted pre-treatment LSC predicted post-SBRT hepatic function decline with 87.5% sensitivity and 72.7% specificity. Jung et al. [21] similarly reported hepatobiliary phase hypointensity in irradiated regions, with a median D_50%_ of 19.8 Gy.

### 3.6 Other imaging findings: MR elastography and morphologic changes

Additional findings further characterized FLR-related parenchymal remodeling. Using magnetic resonance elastography, Omiya et al. [18] reported increased liver stiffness in regions receiving >30 Gy (*p* < 0.05), with no significant changes in lower-dose regions. Morphologic changes were common, particularly capsular retraction: Oldrini et al. [15] reported capsular retraction in 57.1% by 1 year, more frequently in subcapsular lesions (*p* = 0.016), and Serafini et al. [17] observed an increase from 33% to 64% over follow-up (*p* = 0.006). Mendiratta-Lala et al. [14] described delayed enhancement and capsular retraction in adjacent liver parenchyma at 6–12 months, following an earlier phase of hyperemia at 3–6 months.

### 3.7 Tumor size, correlation with treatment response, and reporting of isodose parameters

Serafini et al. [17] reported a mean baseline tumor diameter of approximately 17 mm, which decreased to about 10 mm at the first and second MRI follow-up examinations. Similarly, Mendiratta-Lala et al. [14] observed tumor shrinkage in most treated lesions, with 9 of 10 hepatocellular carcinomas demonstrating a reduction in size within the first year after SBRT. Mai et al. [16] described a gradual decrease in tumor size with stabilization of imaging findings approximately 6–9 months after treatment, whereas lesions with local progression showed interval growth accompanied by persistent arterial phase hyperenhancement and washout.

Baseline tumor size was also associated with treatment outcomes in some studies. Oldrini et al. reported higher response rates for tumors smaller than 20 mm according to RECIST and mRECIST criteria (*p* = 0.034). Similarly, Yu et al. [19] identified primary tumor size as a prognostic factor for local PFS, with tumors ≤3 cm showing significantly better outcomes than larger lesions (1-year LPFS: 100% vs. 79.1%, *p* = 0.01).

Radiotherapy planning parameters were reported inconsistently across the studies. Oldrini et al. [15] indicated that SBRT was delivered with a prescription dose of 45 Gy to the 80% isodose line in three fractions, whereas the other studies mainly reported total dose and fractionation without specifying the prescription isodose level. None of the included studies evaluated potential correlations between isodose distribution and tumor response or imaging findings.

### 3.8 Tumor response versus focal liver reaction

In clinical interpretation, findings are more consistent with FLR when enhancement shows a geographic, rim-like, or perilesional distribution, follows the treated area, and remains unchanged or decreases over time on follow-up MRI. Additional features supporting radiation-related change include stable or reduced lesion size, declining T2-weighted and DWI intensity, increasing ADC values, hepatobiliary phase hypointensity of the adjacent liver parenchyma, and delayed morphologic remodeling such as capsular retraction or focal volume loss. By contrast, residual or recurrent tumor should be considered when enhancement is newly developing, progressive, nodular, or mass-like, especially if accompanied by washout, interval tumor enlargement, increasing T2-weighted or DW signal intensity, lack of ADC increase, or concordant biochemical or clinical evidence of progression. Thus, differentiation between FLR and viable tumor should rely on serial multiparametric MRI assessment [14], [15], [16], [17], [18], [19], [20], [21] (Table 4).

**Table 4:** Expected temporal evolution of MRI findings in treated hepatocellular carcinoma and the surrounding irradiated liver parenchyma at approximately 3, 6, 9, and 12 months after stereotactic body radiation therapy. Findings represent typical imaging patterns and may vary among patients. ADC, apparent diffusion coefficient; APHE, arterial-phase hyperenhancement; DWI, diffusion-weighted imaging; HCC, hepatocellular carcinoma; MRI, magnetic resonance imaging; SBRT, stereotactic body radiation therapy.

Time point	Treated lesion	Surrounding irradiated liver parenchyma	Interpretation
∼3 months	APHE and/or washout may persist, although an early reduction in APHE can occur. Size reduction is often limited. Diffusion restriction may persist, decrease, or be absent.	Focal liver reaction may manifest as ring-like or perilesional enhancement, T2 hyperintensity, and early geographic hepatobiliary-phase hypointensity surrounding the treated lesion.	Persistent early APHE should not be equated with viable tumor or treatment failure. The first post-treatment examination should serve as a baseline for subsequent comparisons, and findings should be interpreted in the clinical and multiparametric imaging context.
∼6 months	APHE and washout often decrease, with evolution toward delayed or absent enhancement. T2- and DWI-related signal abnormalities may decrease, while ADC values tend to increase in responding lesions.	Focal liver reaction and hepatobiliary-phase hypointensity may persist. Delayed perilesional enhancement and early capsular retraction may develop.	Imaging findings commonly begin to stabilize between 6 and 9 months. Multiparametric and longitudinal assessment is preferable to evaluation based on arterial-phase enhancement alone.
∼9 months	Treatment response is usually more established, with further reduction in APHE, stable delayed or absent enhancement, lower T2/DWI signal, and higher ADC values.	Perilesional enhancement and T2 signal abnormalities may persist but are typically stable or less conspicuous. Contraction and atrophy of the surrounding irradiated parenchyma may become apparent.	Stable or regressing perilesional abnormalities favor radiation-related change. New or increasing nodular APHE, particularly when accompanied by washout or lesion growth, should raise suspicion for local tumor progression.
∼12 months	Responding lesions commonly demonstrate absent enhancement or stable delayed enhancement, with lesion size plateauing or continuing to decrease. Small lesions may become inconspicuous or no longer visible.	Late remodeling may include capsular retraction, focal volume loss, and persistent hepatobiliary-phase hypointensity. Retraction and atrophy may be accompanied by crowding or dilatation of peripheral bile ducts.	New or enlarging nodular or mass-like enhancement, recurrent washout, or progressive lesion growth should raise suspicion for local tumor progression.

APHE: arterial phase hyperenhancement; DWI: diffusion weighted imaging; ADC: apparent diffusion coefficient

### 3.9 Role of AFP and longitudinal follow-up assessment

The use of AFP for evaluating treatment response after SBRT varied among the included studies. In the retrospective study by Mendiratta-Lala et al. [14], treatment response was defined either by explant pathology demonstrating >90% tumor necrosis or, in patients without histopathologic confirmation, by biochemical response defined as normalization of elevated pretreatment AFP levels within one year after SBRT (from >75 ng/mL to <10 ng/mL) in the absence of additional therapy. Accordingly, patients either showed >90% tumor necrosis on pathology or a marked decline in AFP levels, with AFP normalization occurring approximately three months after SBRT.

Mai et al. [16] also investigated the relationship between AFP levels and treatment response. Among the 96 patients without local tumor progression, AFP evaluation was performed in a subgroup of 72 patients who had a solitary lesion and no intrahepatic or extrahepatic disease progression during follow-up. Within this subgroup, 30 patients had elevated AFP levels at baseline (>25 IU/mL). Following SBRT, AFP levels decreased substantially, with the most pronounced reduction occurring within the first three months. The median decline was 96.5%, and all patients with elevated baseline AFP values eventually reached normalization during follow-up, with a median time to normalization of three months (range 1–15 months). By contrast, six patients experienced local tumor progression during follow-up. In two cases, progression was confirmed histopathologically after surgical resection, whereas the remaining four cases were diagnosed on the basis of imaging findings in combination with AFP measurements. Imaging indicators of progression included interval lesion enlargement and the presence of persistent or recurrent APHE with washout.

In the remaining studies, AFP was reported mainly as a baseline clinical characteristic rather than as a parameter for response assessment. In the study by Oldrini et al. [15], AFP levels were documented descriptively at baseline, whereas treatment response and follow-up evaluation were based primarily on serial MRI examinations. Similarly, Yu et al. [19] examined AFP as a baseline clinical variable but did not find a significant association between AFP levels and local PFS. The studies by Serafini et al. [17] and Omiya et al. [18] relied predominantly on imaging findings for the evaluation of treatment response, and AFP was not included in the assessment of post-treatment outcomes.

### 3.10 mRECIST versus LI-RADS treatment response assessment after SBRT

After SBRT, treatment response classification may differ depending on the framework applied. mRECIST is a quantitative response system in which viable tumor is defined as the intratumoral arterial-enhancing component, with response determined by changes in the summed diameters of viable target lesions and integrated with the assessment of nontarget lesions and new lesions. This approach may underestimate early response after radiation because APHE can persist temporarily despite effective treatment and local tumor control. In contrast, the LI-RADS CT/MRI Radiation Treatment Response Algorithm v2024 provides a lesion-level assessment specifically adapted to radiation-based therapies and emphasizes the morphology and temporal evolution of enhancement. Absence of masslike enhancement, including complete lesion disappearance, absent lesional enhancement, smooth perilesional enhancement, or geographic nonmasslike parenchymal enhancement, is categorized as LR-TR Nonviable. Masslike enhancement that remains stable or decreases over time is categorized as LR-TR Nonprogressing, whereas new or increasing masslike enhancement is categorized as LR-TR Viable. On MRI, new or increasing diffusion restriction or mild-to-moderate T2 hyperintensity may optionally support upgrading an observation from LR-TR Nonprogressing to LR-TR Viable. This temporal and morphology-based approach is particularly relevant after SBRT, because persistent enhancement and radiation-induced focal liver reaction may otherwise mimic residual disease. LI-RADS Radiation TRA may therefore provide a more tailored framework for post-SBRT assessment, although further validation in SBRT-treated HCC remains warranted [8], [9], [10], [11], [12].

## 4 Discussion

Across the included studies, SBRT was associated with a relatively consistent, time-dependent spectrum of MRI changes in both treated HCC lesions and the surrounding liver parenchyma. Imaging features suggestive of response included a gradual attenuation of APHE and washout, increasing T1-weighted signal intensity, and decreasing T2-weighted and diffusion-weighted signal with rising ADC values, with many changes stabilizing around 6–9 months.

Conversely, persistent APHE and washout in combination with increasing T2-weighted and DWI signal were most often observed in lesions with local progression. Importantly, several studies also highlighted a key interpretive pitfall: isolated persistent enhancement-particularly early after treatment-may occur despite favorable clinical or pathological correlates and in the absence of interval growth or biochemical relapse. These observations reinforce that post-SBRT assessment requires a longitudinal, multiparametric approach rather than reliance on a single time point or arterial-phase criteria alone.

Diffusion imaging may provide complementary, earlier biomarkers of treatment effect. Multiple studies reported increasing ADC values after SBRT, and one study suggested that an ADC increase ≥20% was associated with improved local progression-free survival [19]. While these findings support DWI as a sensitive indicator of post-treatment tissue change, thresholds and effect sizes vary across studies, and clinical implementation will require standardized acquisition and validation in larger cohorts.

Beyond the treated lesion, radiation-induced changes in adjacent liver parenchyma were frequent and can closely mimic residual or recurrent tumor. Reported manifestations of focal liver reaction included ring-like arterial enhancement, perilesional T2 hyperintensity, hepatobiliary phase hypointensity, increased stiffness on MR elastography, and capsular retraction. Hepatobiliary phase imaging, in particular, demonstrated dose-dependent functional impairment in irradiated liver regions, and liver–spleen contrast metrics showed potential utility for predicting post-treatment hepatic function decline. Taken together, the current literature underscores the importance of integrating enhancement behavior with diffusion metrics and hepatobiliary phase findings—and interpreting all features in the context of expected temporal evolution-to reduce misclassification after SBRT.

In clinical interpretation, findings are more consistent with FLR when enhancement shows a geographic, rim-like, or perilesional distribution, follows the treated area, and remains unchanged or decreases over time on follow-up MRI. Additional features supporting radiation-related change include stable or reduced lesion size, declining T2-weighted and DWI intensity, increasing ADC values, hepatobiliary phase hypointensity of the adjacent liver parenchyma, and delayed morphologic remodeling such as capsular retraction or focal volume loss. By contrast, residual or recurrent tumor should be considered when enhancement is newly developing, progressive, nodular, or mass-like, especially if accompanied by washout, interval tumor enlargement, increasing T2-weighted or diffusion-weighted signal, lack of ADC increase, or concordant biochemical or clinical evidence of progression. Thus, differentiation between FLR and viable tumor should rely on serial multiparametric MRI assessment [14], [15], [16], [17], [18], [19], [20], [21].

The evidence base remains limited. Most studies were retrospective and single-center, restricting generalizability and increasing susceptibility to selection bias, particularly where inclusion depended on the availability of follow-up MRI. Reference standards were most commonly based on imaging follow-up and clinical outcomes, while histopathologic correlation was uncommon. Future studies incorporating explant pathology, biopsy confirmation when feasible, or well-defined composite reference standards could improve biological interpretability and strengthen differentiation of residual viable tumor from radiation-induced change.

Interpretation is further complicated by heterogeneity in follow-up schedules. Early post-treatment imaging is particularly prone to false-positive classification of residual disease, underscoring the need for more standardized post-SBRT MRI time points (e.g., 3, 6, 9, and 12 months) to capture the delayed evolution of both tumor response and parenchymal reaction. In parallel, emerging reporting standards—most notably the updated 2024 LI-RADS treatment response algorithm with specific guidance for radiation-based locoregional therapies-aim to improve the consistency and standardization of post-radiation response assessment [22]. Nevertheless, persistent APHE and washout are not uniformly indicative of viability after SBRT, and longitudinal, multiphasic assessment appears more appropriate than reliance on arterial-phase–only criteria.

Several additional gaps warrant attention. DWI and ADC metrics are constrained by variable acquisition protocols and inconsistent thresholds; standardization and validation of reproducible cutoffs are needed. Dose-dependent functional alterations-particularly on hepatobiliary phase imaging-have not been fully characterized, and voxel-based dose–function mapping may improve prediction of regional hepatic dysfunction and enable more individualized functional liver assessment. Advanced quantitative techniques (e.g., MR elastography, intravoxel incoherent motion imaging, and radiomics) remain underrepresented but merit prospective evaluation for noninvasive assessment of radiation-induced liver injury. Finally, established response frameworks, including LI-RADS treatment response algorithm, have not yet been sufficiently validated against SBRT-specific imaging patterns. SBRT-adapted or SBRT-validated response criteria, coupled with correlation to clinically meaningful endpoints (local control, liver function, survival, and toxicity), are needed to support standardized reporting and reduce interobserver variability (Table 4).

## 5 Conclusion

This scoping review maps the spectrum and temporal evolution of MRI findings after SBRT for HCC, highlighting both treatment-related tumor changes and FLR, which can mimic viable or recurrent disease. However, substantial heterogeneity in study design, MRI acquisition, follow-up timing, and reference standards limits comparability across studies. Future prospective multicenter studies should use standardized MRI protocols and predefined follow-up intervals, ideally including assessments at approximately 3, 6, 9, and 12 months after SBRT. Such harmonization, together with validation of contemporary response frameworks (e.g., LI-RADS v2024 guidance for radiation-based therapies), is needed to improve reproducibility, reduce misclassification, and support more consistent post-SBRT response assessment.

## CRediT authorship contribution statement

**Caglayan Lara:** Writing – review & editing, Writing – original draft, Data curation. **Gkika Eleni:** Writing – review & editing, Writing – original draft. **Péporté Anne:** Writing – review & editing. **Stocker Daniel:** Writing – review & editing, Writing – original draft, Validation, Supervision, Methodology. **Ghafoor Soleen:** Writing – review & editing, Writing – original draft, Visualization, Validation, Supervision, Project administration, Methodology, Formal analysis, Conceptualization.

## Funding

None

## Declaration of competing interest

The authors declare that they have no known competing financial interests or personal relationships that could have appeared to influence the work reported in this paper.
